# Improvement in nasal signs and eosinophils following 3 years of sublingual immunotherapy for allergic rhinitis

**DOI:** 10.3389/fimmu.2026.1828695

**Published:** 2026-04-28

**Authors:** Beina Liu, Sunhong Hu, Mang Xiao

**Affiliations:** Department of Otolaryngology-Head and Neck Surgery, Sir Run Run Shaw Hospital, Medical College of Zhejiang University, Hangzhou, China

**Keywords:** allergic rhinitis, efficacy, endoscopy, eosinophil, sublingual immunotherapy

## Abstract

**Background:**

While sublingual immunotherapy (SLIT) is effective for allergic rhinitis (AR), most studies focus on subjective symptom relief, with limited data on objective signs and biomarkers. This study evaluates the effects of 3-year house dust mite (HDM) SLIT on nasal mucosal signs and peripheral blood eosinophil (EOS) counts in AR patients.

**Methods:**

This study retrospectively analyzed the data of 86 AR patients (aged 14–57) who had received 3 years of SLIT treatment. Subjective scores including the total nasal symptom score (TNSS), total medication score (TMS), combined symptom and medication score (CSMS), and visual analog scale (VAS) were assessed at baseline, 1.5 years, and 3 years. Objective assessment of nasal mucosal pathology was performed via nasal endoscopy and quantified using the modified Lund-Kennedy (MLK) scoring system. Additionally, EOS counts and the level of serum total IgE were also measured.

**Results:**

At 1.5 and 3 years, significant reductions were observed in TNSS, TMS, CSMS, VAS, and MLK scores compared to baseline and between these timepoints (all *p* < 0.001). Nasal endoscopy at the 3-year follow-up revealed marked improvement in nasal mucosal edema and secretions. After the 3-year treatment, EOS counts significantly decreased (*p* < 0.001), whereas total IgE levels remained unchanged. Furthermore, the overall clinical improvement rate in this study reached 96.51%. No serious adverse events (AEs) were reported during the whole study.

**Conclusion:**

Three years of SLIT treatment effectively improved symptoms of HDM-induced allergic rhinitis, significantly reduced signs of nasal mucosal inflammation, and decreased peripheral blood eosinophil counts.

## 1 Introduction

Allergic rhinitis (AR) is a chronic inflammatory disease of the nasal mucosa mediated by immunoglobulin E (IgE), affecting approximately 10% to 40% of the global population. Although its prevalence in China is slightly lower than in developed countries, it is estimated that around 119 million individuals (including 89.74 million adults and 29 million children) are affected (1, 2). Notably, the overall prevalence of AR continues to increase globally. AR patients frequently experience symptoms such as nasal congestion, pruritus, repetitive sneezing, and rhinorrhea, which significantly impair cognitive function, quality of life and sleep. AR may also be accompanied by comorbidities such as chronic rhinosinusitis, asthma, and sleep apnea syndrome (3, 4). The pathophysiology of AR primarily involves a dysregulated type II immune response, leading to the production of allergen-specific IgE. Inflammatory cells, including mast cells, basophils, and eosinophils (EOS), are recruited and infiltrate the nasal mucosa, releasing abundant pro-inflammatory mediators that perpetuate allergic inflammation and elicit clinical symptoms (5, 6). Allergen-specific immunotherapy (AIT) has a development history spanning over a century and remains the only etiological treatment capable of modifying the natural course of allergic diseases (7). Among its methods, sublingual immunotherapy (SLIT) has emerged as a primary therapeutic option for AR owing to its favorable safety profile and convenient administration (8–10). SLIT involves the repeated sublingual administration of standardized allergen extracts over an extended period to induce and maintain allergen-specific immune tolerance. Key immunological mechanisms include promoting the activation of regulatory T cells (Treg) and regulatory B cells (Breg), as well as stimulating the production of blocking antibodies IgG4. These responses collectively contribute to the suppression of Th2-driven inflammatory pathways and facilitate the restoration of immunological homeostasis (5, 11, 12). Previous studies (13–15) have established that Dermatophagoides farinae (*D. farinae*) drops can effectively alleviate the clinical symptoms and reduce the use of rescue medications in AR patients. However, these assessments have relied primarily on patients’ subjective descriptions, such as the total nasal symptom score (TNSS) and visual analog scale (VAS). Conversely, objective evaluation methods, particularly endoscopic findings assessed using the modified Lund-Kennedy (MLK) scoring systems, have been underutilized. This gap has resulted in a lack of direct and objective evidence regarding structural improvements in nasal mucosa following immunotherapy. In terms of biomarkers, allergen-specific IgE and IgG4 are well-established potential indicators that reflect the efficacy of AIT. Meanwhile, eosinophils, which are multifunctional leukocytes and the primary effector cells in the allergic process (16), serve as inflammatory biomarkers of AR. In AR, the degree of local eosinophil infiltration in the nasal mucosa is closely associated with disease severity, and peripheral blood eosinophil counts, owing to their ease of measurement and high accuracy, have been widely used as objective indicators for monitoring allergic responses and evaluating treatment efficacy (17, 18). Nonetheless, reports on their utility in reflecting AIT efficacy remain relatively limited, and their dynamic changes over the course of long-term allergen immunotherapy remain insufficiently characterized. Additionally, total IgE (tIgE) can serve as a non−specific indicator of allergic reactions, as noted in the sublingual immunotherapy: World Allergy Organization (WAO) position paper (19). Therefore, this study aimed to quantitatively assess the improvement of nasal signs from an objective perspective to evaluate the efficacy of house dust mite (HDM) SLIT with *D. farinae* drops. Furthermore, we investigated the changes in peripheral blood eosinophil counts and tIgE levels following three years of SLIT treatment to provide a more comprehensive basis for its clinical application.

## 2 Materials and methods

### 2.1 Study subjects

This clinical study was approved by the Ethics Committee of Sir Run Run Shaw Hospital, Medical College of Zhejiang University and was conducted in accordance with the principles of the Declaration of Helsinki. In this retrospective study, a total of 86 patients with AR who were admitted to our hospital from October 2017 to September 2022 and received SLIT treatment for 3 years were included, aged 14 to 57 years. All the included patients met the SLIT treatment criteria, as detailed below: (1) All patients were diagnosed with AR based on Allergic Rhinitis and its Impact on Asthma (ARIA) guidelines (20); (2) Patients had a history of dust mite allergy and sensitization to *D. farinae* with/without *Dermatophagoides pteronyssinus* (*D. pteronyssinus*), and were evaluated as positive (≥++) by skin prick test or with a serum sIgE detection of ≥0.35 KU/L. The interpretation of skin prick test results was conducted strictly in accordance with the kit instructions: a result was considered positive when skin wheal areas produced by allergen extracts were over 25% of the areas produced by the positive control. On this basis, further grading was performed according to the skin wheal area ratio: “+” = 26%~50%, “++” = 51%~100%, “+++” = 101%~200%, and “++++” = over 200%; (3) Patient without tumors, autoimmune diseases, significant organ dysfunction, current pregnancy or lactation, and patients who have taken β blockers or angiotensin-converting enzyme inhibitors.

### 2.2 Treatment schedule

All patients underwent SLIT using standardized *D. farinae* drops (Chanllergen, Zhejiang Wolwo Bio-pharmaceutical Co., Ltd., China), with the treatment period divided into the incremental phase and maintenance phase. *D. farinae* drops had 5 specifications, and the concentrations are increasing successively. The total protein concentrations corresponding to No. 1 to No. 5 were 1, 10, 100, 333, and 1000 μg/ml, respectively, determined using the bicinchoninic acid (BCA) protein assay. According to the instruction of the drops, patients used No. 1 to No. 3 in the incremental stage of the first 3 weeks, and the number of drops used on the 1st to 7th day of each week were 1, 2, 3, 4, 6, 8, and 10 drops, respectively. During the 4th to 5th weeks, patients used 3 drops of No. 4 daily. From week 6 until treatment completion, maintenance therapy consisted of 2 drops of No. 5 daily. The drops should be administered sublingually, and retained 1–3 minutes before swallowing. Daily administration at the same time is recommended. The first dose required 30 minutes of observation at the hospital. In this study, all patients received appropriate symptomatic medications as needed during SLIT treatment, in accordance with the ARIA guidelines (20). When symptoms improved, step-down therapy was applied for symptomatic treatment.

### 2.3 Efficacy evaluation

The first-visit record data of patients at the hospital, such as nasal symptoms, medication use, and severity were taken as baseline data. Subsequently, TNSS, total medication score (TMS), combined symptom and medication score (CSMS), and VAS scores of the patients after 1.5 years and 3 years of treatment were analyzed based on the follow-up records. Additionally, according to the results of nasal endoscopy examinations at these three time points, the changes in the patients’ nasal mucosa were evaluated and graded using the MLK scoring system. The outcome indicators are as follows:

TNSS refers to the sum of the scores of four nasal symptoms (nasal congestion, nasal itching, sneezing and discharge), which are scored on a scale of 0 to 3 (0 = no symptoms; 1 = mild symptoms that were easy to tolerate; 2 = obvious symptoms, annoying, but tolerable; 3 = unbearable symptoms that affected daily life and/or sleep), ranging from 0 to 12 (21).

TMS was recorded ranging from 0 to 3 points (0 = no medicine, 1= oral antihistamine or anti-leukotriene, 2 = nasal glucocorticoid, 3 = oral glucocorticoid).

CSMS is an aggregative scoring indicator for patients’ symptoms and medications, with the calculation formula: CSMS=TNSS/4+TMS, ranging from 0 to 6.

The severity of the patient’s symptoms is reflected by the VAS score, with a 10cm scale representing a score range of 0 to 10 (0 = no symptoms, and 10 = the most severe symptoms). Patients were categorized as mild, moderate, or severe based on the VAS score: 1–3 points as mild, 4–7 points as moderate, and 8–10 points as severe (22).

The MLK endoscopic score is the sum of the three components of nasal polyps, secretions, and mucosal edema, each on a 3-point scale of 0 to 2 (23): (1) nasal polyps (none = 0, confined to middle meatus = 1, beyond middle meatus = 2); (2) secretions (none = 0, clear and thin = 1, viscous and purulent = 2); (3) edema (absent = 0, mild = 1, severe = 2). In this study, the patients’ nostrils on both sides were scored separately, and the mean was calculated. Additionally, two patients were randomly selected to analyze the results of nasal endoscopy pre- and post-treatment.

Furthermore, this study analyzed immunological parameters in patients before and after treatment. Due to testing costs and compliance, 45 patients (52%) underwent serum total IgE level and EOS counts examination for evaluating the impact of 3 years SLIT on immune function in AR patients.

Patients were categorized based on their CSMS reduction rate into the following groups for clinical efficacy assessment: well improved group (ΔCSMS ≥ 50%), partially improved group (20% <ΔCSMS < 50%), unimproved group (ΔCSMS ≤ 20%) (24).

### 2.4 Safety

Safety was analyzed based on the adverse events (AEs) recorded during the study period. All AEs were addressed under the recommendation of the physicians.

### 2.5 Statistical analysis

The statistical analysis was performed with SPSS version 20.0 software (SPSS, Inc., Chicago, IL, USA) with a 5% significance level. Continuous variables were shown as mean ± standard deviation (SD). For repeated comparison across multiple time points (baseline, 1.5 years, and 3 years) of indicators such as TNSS, TMS, VAS, CSMS, and MLK scores, the Friedman test was used for overall comparison, followed by adjusted post−hoc pairwise analyzes using the Dunn−Bonferroni correction. For comparisons between two time points, such as eosinophil counts and tIgE levels, the Wilcoxon signed−rank test was applied. The chi-square test is used to analyze categorical variables. *p* < 0.05 represented significant difference.

## 3 Results

### 3.1 Subjects characteristics

**Figure 1:**
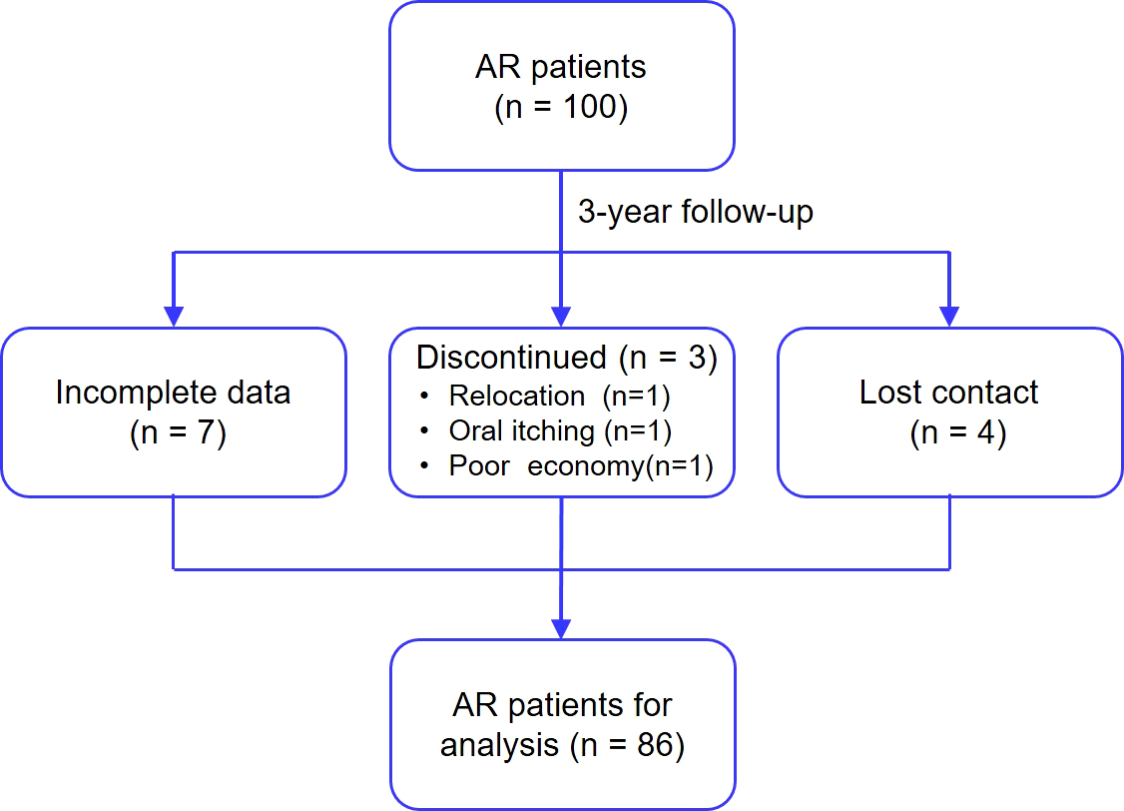
Flowchart of patient enrollment. AR, allergic rhinitis; SLIT, sublingual immunotherapy.

A cohort of 86 patients aged 14 to 57 years (mean age: 29.58 ± 9.99 years) with HDM-induced AR were enrolled in this study (**Figure 1**), comprising 34 males (39.5%) and 52 females (60.5%). The demographic characteristics were shown in **Table 1**.

**Table 1:** The baseline characteristics of the patients.

Characteristics	Patients
Numbers of patients	86
Age (years), Mean ± SD^*^	29.58 ± 9.99
Gender	
Male (n, %)	34 (39.5)
Female (n, %)	52 (60.5)
Clinical scores (Mean ± SD)	
Baseline TNSS^†^	8.10 ± 1.82
Baseline TMS^‡^	1.98 ± 0.21
Baseline CSMS^§^	4.00 ± 0.48
Baseline VAS^∥^	7.36 ± 1.63
Baseline MLK^¶^	2.34 ± 0.95

* SD, standard deviation.

^†^TNSS, total nasal symptom score.

^‡^TMS, total medication score.

^§^CSMS, combined symptom and medication score.

^∥^VAS, visual analog scale.

^¶^MLK, modified Lund-Kennedy.

### 3.2 Clinical evaluation on TNSS, TMS, CSMS and VAS

**Figure 2:**
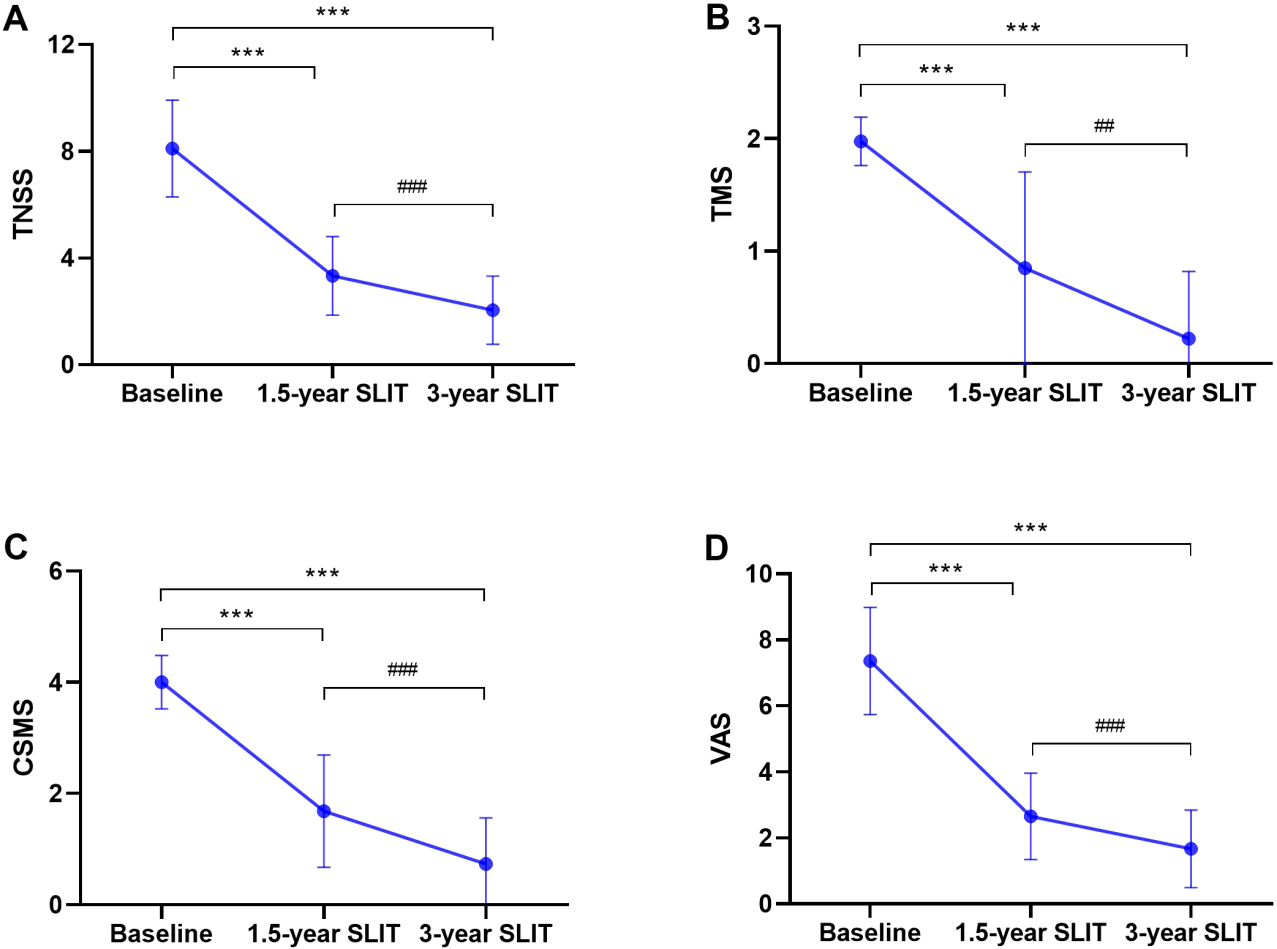
Variations in TNSS **(A)**, TMS **(B)**, CSMS **(C)**, and VAS **(D)** scores at baseline, 1.5 years, and 3 years of SLIT treatment. TNSS:total nasal symptom score; TMS:total medication score; CSMS: combined symptom and medication score; VAS: visual analog scale; SLIT: sublingual immunotherapy. (^***^p < 0.001, compared with baseline; ^###^*p* < 0.001, compared with 1.5 years of SLIT treatment.).

The changes in TNSS, TMS, CSMS, and VAS scores of the patients before and after SLIT treatment were presented in **Figure 2**. Compared with baseline, patients’ TNSS, TMS, CSMS, and VAS scores decreased significantly after 1.5 years and 3 years of SLIT (8.10 ± 1.82, 1.98 ± 0.21, 4.00 ± 0.48, and 7.36 ± l.63 at baseline vs. 3.34 ± 1.47, 0.85 ± 0.86, 1.68 ± 1.01, and 2.65 ± 1.31 at 1.5 years vs. 2.05 ± 1.27, 0.22 ± 0.60, 0.73 ± 0.83, and 1.67 ± 1.17 at 3 years, all *p* < 0.001). Furthermore, all four subjective scores showed a continued significant decline after 3 years of treatment compared to 1.5 years (all *p* < 0.01).

### 3.3 Clinical evaluation on MLK scoring system and nasal endoscopy observation

**Figure 3:**
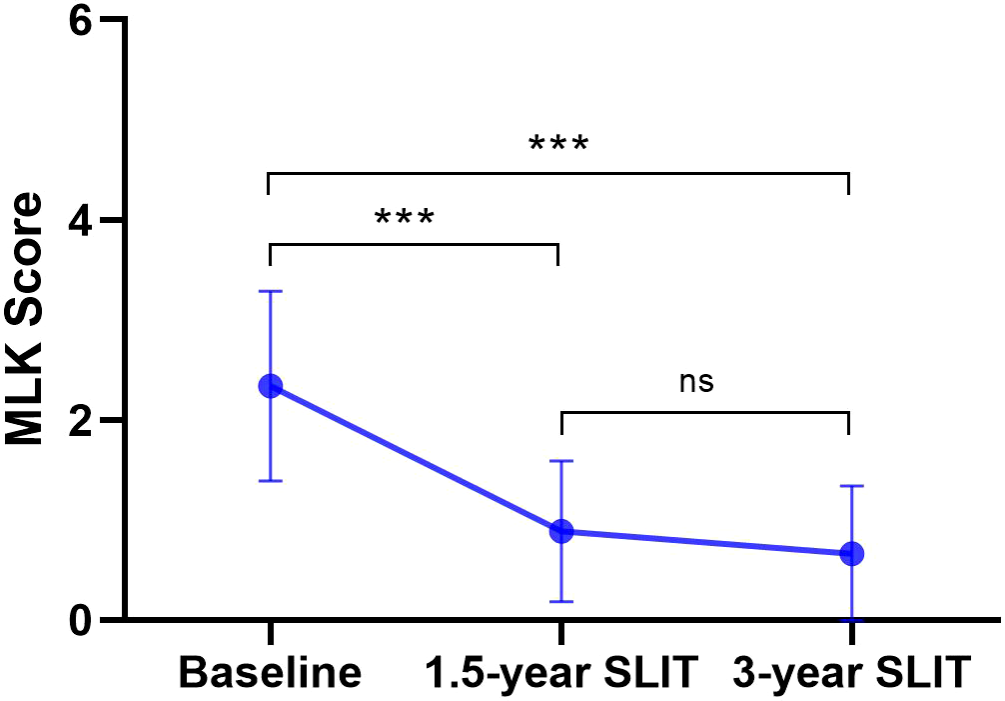
Variations in MLK score at baseline, 1.5 years, and 3 years of SLIT treatment. MLK, modified Lund-Kennedy; SLIT, sublingual immunotherapy. (^***^p < 0.001, compared with the baseline; ^#^*p* < 0.05, compared with the 1.5 years of SLIT treatment.).

Patients in this study underwent nasal endoscopy examinations at baseline, 1.5 years, and 3 years of follow-up, and the results were shown in **Figure 3**. The mean MLK scores at baseline, 1.5 years, and 3 years of SLIT were 2.34 ± 0.95, 0.88 ± 0.70, and 0.66 ± 0.67, respectively. Similar to the trend of TNSS score, the MLK scores declined dramatically at both 1.5 and 3 years compared to baseline (both *p* < 0.001). Moreover, no statistically significant difference was observed in MLK scores between the 1.5−year and 3−year time points (*p* > 0.05), while the mean MLK score at 3 years was lower than that at 1.5 years, indicating that the improvement in nasal mucosa may have been stably maintained during the long−term treatment period.

**Figure 4:**
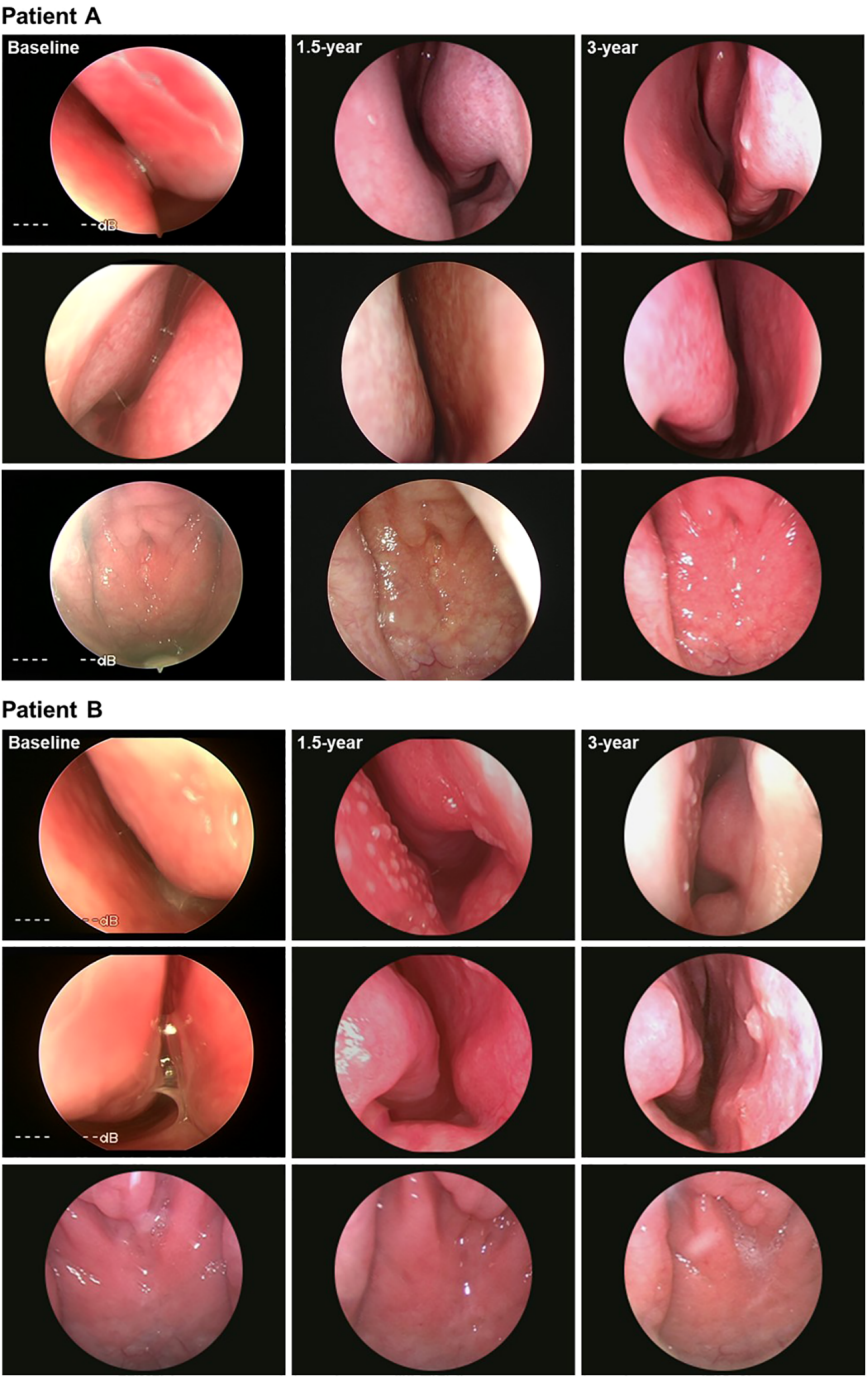
Representative endoscopic findings at baseline, 1.5 years, and 3 years of SLIT in 2 patients (Patient **A** and Patient **B**) with AR. AR: allergic rhinitis; SLIT: sublingual immunotherapy. The images from top to bottom represented the mucosa of the left nostril, the mucosa of the right nostril and the nasopharynx in sequence.

Based on the aforementioned nasal endoscopic examination, this study analyzed nasal endoscopic images from two randomly selected cases, as shown in **Figure 4**. At baseline, patient A showed edema and congestion of the nasal mucosa in both nostrils, accompanied by secretory effusion. After 3 years of SLIT, the mucosal surface became smoother and slightly drier, more closely resembling normal mucosa, with a noticeable reduction in secretions. Moreover, the color of the nasopharyngeal mucosa changed from pale at baseline to pink after treatment. Patient B exhibited similar improvements in mucosal edema, color change, and secretion reduction. Compared to baseline, both cases demonstrated significant improvement in nasal endoscopic findings after treatment.

### 3.4 Serum total IgE and eosinophil count levels

**Figure 5:**
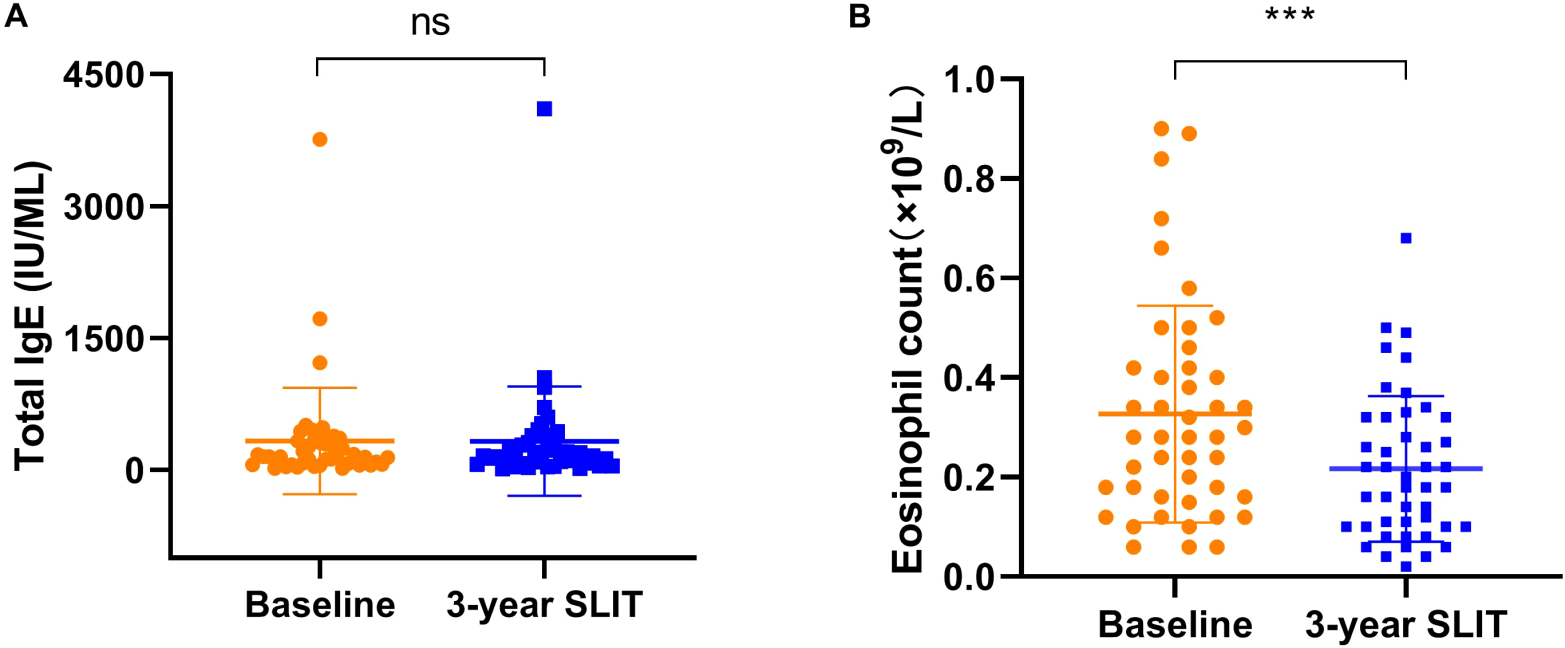
Variations in serum total IgE **(A)** and Eosinophil counts **(B)** at baseline and after 3 years of SLIT treatment in some patients. SLIT, sublingual immunotherapy (^***^p < 0.001, compared with the baseline.).

The total serum IgE and peripheral blood eosinophil counts of some patients before and after treatment were shown in **Figure 5**. The serum total IgE levels were 332.10 ± 597.65 IU/mL at baseline and 328.35 ± 613.45 IU/mL after treatment, showing no significant change following therapy (*p* > 0.05). In contrast, the eosinophil counts were 0.33 ± 0.21 (×10^9^/L) and 0.21 ± 0.14 (×10^9^/L) at baseline and after 3 years of SLIT, respectively, representing a substantial reduction after treatment (*p* < 0.001).

### 3.5 Degree of clinical improvement

**Figure 6:**
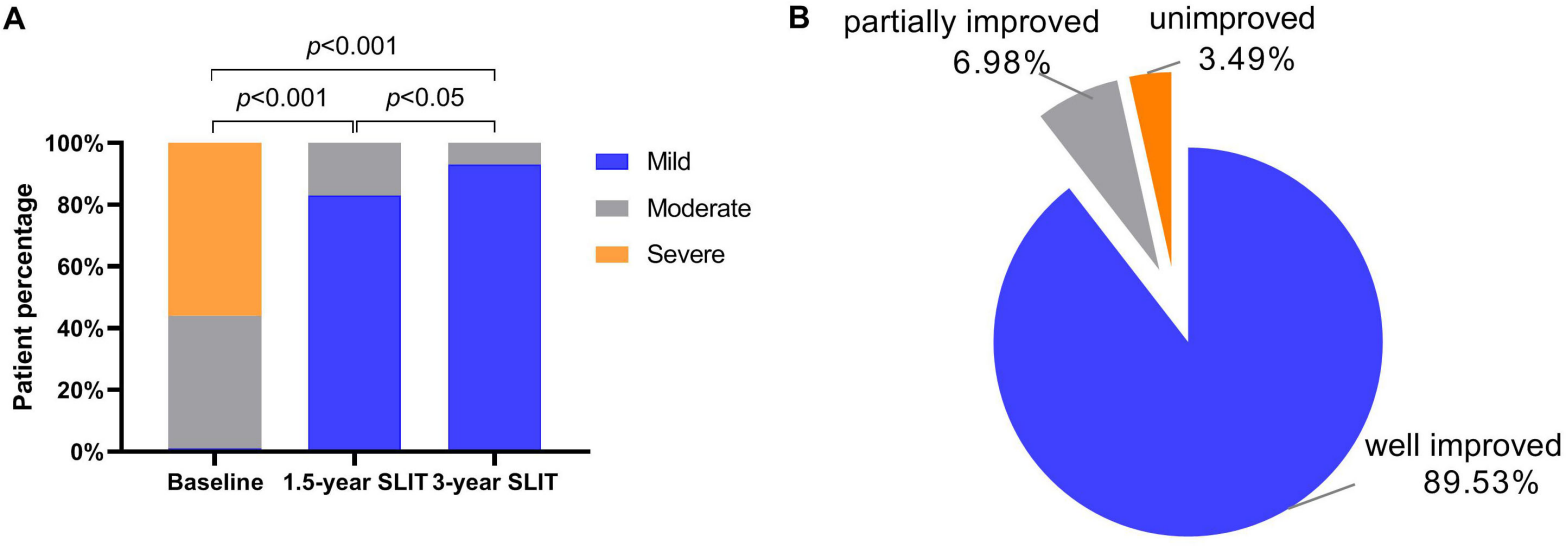
**(A)** Severity of patients’ symptoms at baseline, 1.5 years, and 3 years of SLIT treatment; **(B)** Improvement rate of allergic rhinitis patients after 3 years of SLIT treatment. SLIT, sublingual immunotherapy.

As depicted in **Figure 6A**, 98.8% of the patients were classified as moderate to severe patients at baseline, among which 43% were moderate and 55.8% were severe. However, after 1.5 years of SLIT, significant clinical improvement was observed: 71 patients (82.5%) were classified as mild and 15 patients (17.5%) as moderate, with no patients remaining in the severe category, which was significantly different from baseline (*p* < 0.001). After 3 years of treatment, further symptomatic improvement was evident, with mild cases increasing to 80 patients (93.02%) and moderate cases decreasing to 6 patients (6.98%), revealing significant improvement compared to both baseline (*p* < 0.001) and 1.5 years (*p* < 0.05).

In addition, the overall improvement of patients was further analyzed based on the reduction rate of CSMS before and after treatment. The incidence rates of well improved and partially improved were 89.53% (77/86) and 6.98% (6/86), respectively, while only 3.49% (3/86) of patients belonged to the unimproved group (**Figure 6B**). In the analysis cohort of this study, the total improvement rate (well improved and partially improved) after SLIT treatment reached 96.51%.

### 3.6 Safety

No serious AEs occurred during the entire treatment period. Among all patients receiving SLIT treatment, a total of 11 patients (12.8%) experienced AEs (**Table 2**). All adverse events were mild to moderate local reactions, mainly presenting as oral numbness, exacerbation of rhinitis, throat itching, and gastrointestinal discomfort. Most adverse events resolved spontaneously within one week without intervention, and only three patients used anti-allergic medication to control symptoms.

**Table 2:** Analysis of adverse events during the study.

Adverse event	Treatment group (n)
Oral numbness	4
Exacerbation of rhinitis	2
Throat itching	3
Gastrointestinal discomfort	2

## 4 Discussion

AR is one of the common chronic diseases worldwide, significantly impairing patients’ quality of life and social functioning. As a disease-modifying treatment, SLIT aims to induce immunological tolerance, providing sustained relief of symptoms and reducing the need for pharmacotherapy (11). With ongoing advancements, SLIT is highly recommended in clinical practice due to its superior safety profile—notably avoiding the risk of severe systemic adverse reactions associated with subcutaneous immunotherapy (SCIT) (25, 26). Currently, standardized SLIT preparations are widely used for AR patients, providing them with a long-term management strategy.

Numerous clinical studies have shown that SLIT is significantly effective in treating HDM-induced AR. In a randomized controlled trial, Cheng et al. (13) reported that patients receiving SLIT for 3 years exhibited significant reductions in both symptom and medication scores compared to baseline. Moreover, there was no significant difference between the 3-year and 6-year follow-up results in the SLIT group. Similarly, Lin et al. (27) explored the clinical effects of different SLIT treatment durations for AR patients, confirming that a 3-year SLIT course was more effective than 1- or 2-year courses and that patients gained a sustained clinical benefit for 1 year after SLIT. In this retrospective study, we evaluated the efficacy of a 3-year HDM SLIT treatment in 86 patients with AR. The results showed significant reductions in TNSS, TMS, CSMS, and VAS scores following the treatment period, consistent with previous published findings. We also analyzed changes in symptom severity before and after treatment using VAS score: all severe cases shifted to either mild or moderate, with mild cases accounting for (93.02%) and moderate cases accounting for (6.98%). Additionally, we referred to the CSMS reduction rate proposed by Gao et al. (24) to assess the degree of disease improvement. The clinical improvement rate observed in our study cohort was 96.51%, which was consistent with the findings reported by Novakova et al. (28). In summary, as the treatment duration was extended to 3 years, all scores continued to improve, thereby suggesting the potential effectiveness of SLIT from a subjective perspective.

The MLK scoring system has been established as an objective tool for assessing inflammatory changes in the nasal mucosa of patients with AR (29, 30). It standardizes the evaluation of nasal polyps, secretions, and mucosal edema, effectively reducing subjective bias. In the present study, nasal endoscopic examination revealed that nasal polyps observed in some patients at baseline were completely resolved after treatment. Furthermore, nasal mucosal edema and secretions were also significantly alleviated. These findings aligned with those previously reported (31) by our group and further supported the potential value of nasal endoscopy in assessing SLIT efficacy. Overall, this study addressed the prior drawback of depending only on patients’ subjective symptom scores by incorporating the MLK scores as one of the primary objective endpoints, thereby providing supportive evidence that long-term SLIT may be associated with reduced local inflammation of the nasal mucosa.

Notably, there were few reports in the literature on the dynamic changes in peripheral blood EOS counts after long-term SLIT treatment. As the principal effector cells of Th2-mediated inflammation, eosinophils play a key role in allergic inflammation within the nasal mucosa and bronchial epithelial tissues (16). Studies have shown that AIT can inhibit peripheral blood eosinophil infiltration and reduce the expression of adhesion molecules in patients (32). Additionally, AIT can downregulate the expression of Th2-type cytokines (such as IL-5) and chemokines (such as eotaxin), thereby suppressing eosinophil recruitment and survival and reducing tissue damage (12, 16). These mechanisms provide a theoretical basis for the decrease in peripheral blood eosinophil counts observed in this study, suggesting that SLIT may reduce eosinophil production and recruitment by suppressing systemic Th2 inflammation, which was in accordance with the objective improvement of nasal mucosal inflammation. This finding aligned with some short-term studies (23, 33), among which Wei et al. (33) reported that the percentage of eosinophils significantly decreased after 12 months of SLIT treatment in AR patients. The findings of this study suggested that three-year SLIT treatment may modulate Th2-type inflammation by reducing eosinophil production, indicating that EOS could serve as a potential inflammatory biomarker for evaluating the efficacy of SLIT. However, EOS alone is insufficient to explore the immune tolerance induced by SLIT treatment, and future studies are required to explore this mechanism more thoroughly.

In addition to eosinophils, we also examined changes in tIgE. In our department’s allergy screening, it remains an auxiliary screening indicator for allergic diseases. Here, tIgE levels were measured in a subset of patients (n=45), and no significant changes were observed after analysis, which is consistent with the majority of published reports (34, 35). This may be because tIgE is a non-specific indicator that reflects the overall atopic status of the body rather than an immune response to specific allergens (19). Therefore, although tIgE can be used as a general screening indicator for allergic diseases, it is not suitable as a sensitive indicator for assessing SLIT efficacy.

SLIT is widely employed in clinical practice due to its good safety profile. In this study, no serious adverse events were recorded during treatment, and the reported safety findings were consistent with those in previous studies (13, 14). All recorded adverse events were mild to moderate and most resolved spontaneously within one week.

This study has certain limitations. First, it employed a retrospective single-arm design without a placebo, medication, or untreated control group, making it difficult to completely exclude the influence of confounding factors on the observed improvements and thereby limiting the strength of causal inference. Second, incomplete biomarker sampling (n=45) limited the statistical power to some extent. Additionally, this study only used peripheral blood eosinophil counts and tIgE as biological inflammatory indicators, which were insufficient to fully reflect whether SLIT treatment induced immune tolerance. Finally, the allergen component information of the SLIT product used in this study is incomplete, which to some extent limits our ability to fully assess the reproducibility across different SLIT products. In view of the above shortcomings, future prospective randomized controlled trials are needed, with placebo or control groups, and multiple immunological parameters such as sIgE, sIgG4, and sIgE/tIgE ratio should be measured, along with detailed characterization of the allergen composition of SLIT preparations, to further validate the clinical findings of this study and investigate the underlying immunological mechanisms.

In summary, a three-year course of SLIT was associated with favorable clinical outcomes in this cohort of patients with AR. The therapy was observed to significantly improve subjective symptom and medication scores, and nasal endoscopic findings suggested its potential to alleviate nasal mucosal inflammation, accompanied by a reduction in peripheral blood eosinophil counts. Thus, the study suggested the potential key role of nasal endoscopy and eosinophil counts in evaluating the efficacy of SLIT, providing a possible objective basis for more accurate monitoring of the therapeutic effect in the future.

## Data Availability

The raw data supporting the conclusions of this article will be made available by the authors, without undue reservation.

## References

[1] Brożek JL Bousquet J Agache I Agarwal A Bachert C Bosnic-Anticevich S . Allergic rhinitis and its impact on asthma (ARIA) guidelines-2016 revision. J Allergy Clin Immunol. (2017) 140:950–8. doi: 10.1016/j.jaci.2017.03.050. PMID: 28602936

[2] Wang N Yao Y Liu YH Zeng M Liu Z . Allergic rhinitis in China: Trends, challenges and implications over the past two decades. Clin Exp Allergy. (2025) 55:648–58. doi: 10.1111/cea.70118. PMID: 40676746

[3] Fawzan AE Assiri SA Althaqafi RMM Alsufyani A Alghamdi ASA . Association of allergic rhinitis with hypothyroidism, asthma, and chronic sinusitis: clinical and radiological features. World J Otorhinolaryngol Head Neck Surg. (2022) 8:262–8. doi: 10.1016/j.wjorl.2020.12.001. PMID: 36159906 PMC9479480

[4] Ferreira NB Ponte A Grande AC Pimenta AC Pinto CS Bousquet J . Frequency of obstructive sleep apnea in patients with asthma or allergic rhinitis: a systematic review and meta-analysis. Sleep Med. (2025) 134:106705. doi: 10.1016/j.sleep.2025.106705. PMID: 40774162

[5] Yang M Sun L Zhu D Meng C Sha J . Recent advances in understanding the effects of T lymphocytes on mucosal barrier function in allergic rhinitis. Front Immunol. (2023) 14:1224129. doi: 10.3389/fimmu.2023.1224129. PMID: 37771581 PMC10523012

[6] Wang J Zhou Y Zhang H Hu L Liu J Wang L . Pathogenesis of allergic diseases and implications for therapeutic interventions. Signal Transduct Target Ther. (2023) 8:138. doi: 10.1038/s41392-023-01344-4. PMID: 36964157 PMC10039055

[7] Jutel M Agache I Bonini S Burks AW Calderon M Canonica W . International consensus on allergy immunotherapy. J Allergy Clin Immunol. (2015) 136:556–68. doi: 10.1016/j.jaci.2015.04.047. PMID: 26162571

[8] Wise SK Damask C Roland LT Ebert C Levy JM Lin S . International consensus statement on allergy and rhinology: Allergic rhinitis - 2023. Int Forum Allergy Rhinol. (2023) 13:293–859. doi: 10.1002/alr.23090. PMID: 36878860

[9] Waserman S Shah A Avilla E . Recent development on the use of sublingual immunotherapy tablets for allergic rhinitis. Ann Allergy Asthma Immunol. (2021) 127:165–175.e1. doi: 10.1016/j.anai.2021.05.020. PMID: 34029713

[10] Yang Y Li W Zhu R . Allergen immunotherapy in China. Front Allergy. (2024) 4:1324844. doi: 10.3389/falgy.2023.1324844. PMID: 38260178 PMC10801290

[11] Głobińska A Boonpiyathad T Satitsuksanoa P Kleuskens M van de Veen W Sokolowska M . Mechanisms of allergen-specific immunotherapy: Diverse mechanisms of immune tolerance to allergens. Ann Allergy Asthma Immunol. (2018) 121:306–12. doi: 10.1016/j.anai.2018.06.026. PMID: 29966703

[12] Zemelka-Wiacek M Agache I Akdis CA Akdis M Casale TB Dramburg S . Hot topics in allergen immunotherapy, 2023: Current status and future perspective. Allergy. (2024) 79:823–42. doi: 10.1111/all.15945. PMID: 37984449

[13] Chen WB Shen XF Li Q Zhou WC Cheng L . Efficacy of a 3-year course of sublingual immunotherapy for mite-induced allergic rhinitis with a 3-year follow-up. Immunotherapy. (2020) 12:891–901. doi: 10.2217/imt-2020-0006. PMID: 32693660

[14] Ma X Zhang Y Gu X Wu G Liu J Lu J . A retrospective cohort study of sublingual immunotherapy with standardized Dermatophagoides farinae drops for allergic rhinitis. Adv Ther. (2021) 38:2315–22. doi: 10.1007/s12325-021-01686-x. PMID: 33740216

[15] Zhang Y Li J Long Y Ling Z . Enhancing quality of life with 3-year course of sublingual immunotherapy for house dust mite-induced allergic rhinitis: An observational prospective study in real-life settings. Am J Otolaryngol. (2024) 45:104418. doi: 10.1016/j.amjoto.2024.104418. PMID: 39067091

[16] Kim CK Callaway Z Park JS Pawankar R Fujisawa T . Biomarkers in allergen immunotherapy: Focus on eosinophilic inflammation. Asia Pac Allergy. (2024) 14:32–8. doi: 10.5415/apallergy.0000000000000129. PMID: 38482456 PMC10932480

[17] Mormile M Mormile I Fuschillo S Rossi FW Lamagna L Ambrosino P . Eosinophilic airway diseases: From pathophysiological mechanisms to clinical practice. Int J Mol Sci. (2023) 24:7254. doi: 10.3390/ijms24087254, PMID: 37108417 PMC10138384

[18] Celis-Preciado CA Lachapelle P Couillard S . Blood eosinophils take centre stage in predicting the response to sublingual immunotherapy (SLIT): a familiar twist. Thorax. (2024) 79:297–8. doi: 10.1136/thorax-2023-221274. PMID: 38359922

[19] Canonica GW Cox L Pawankar R Baena-Cagnani CE Blaiss M Bonini S . Sublingual immunotherapy: World Allergy Organization position paper 2013 update. World Allergy Organ J. (2014) 7:6. doi: 10.1186/1939-4551-7-6. PMID: 24679069 PMC3983904

[20] Brozek JL Bousquet J Baena-Cagnani CE Bonini S Canonica GW Casale TB . Allergic rhinitis and its impact on asthma (ARIA) guidelines: 2010 revision. J Allergy Clin Immunol. (2010) 126:466–76. doi: 10.1016/j.jaci.2010.06.047. PMID: 20816182

[21] Pfaar O Demoly P Gerth van Wijk R Bonini S Bousquet J Canonica GW . Recommendations for the standardization of clinical outcomes used in allergen immunotherapy trials for allergic rhinoconjunctivitis: an EAACI Position Paper. Allergy. (2014) 69:854–67. doi: 10.1111/all.12383. PMID: 24761804

[22] Del Cuvillo A Santos V Montoro J Bartra J Davila I Ferrer M . Allergic rhinitis severity can be assessed using a visual analog scale in mild, moderate and severe. Rhinology. (2017) 55:34–8. doi: 10.4193/Rhin16.025. PMID: 28019644

[23] Zhang Y Jiang H Long Y Li J . The evaluation value of the modified Lund-Kennedy nasal endoscopy score on the efficacy of sublingual immunotherapy for allergic rhinitis. Am J Rhinol Allergy. (2024) 38:366–72. doi: 10.1177/19458924241269786. PMID: 39152637

[24] Gao Y Lin X Ma J Wei X Wang Q Wang M . Enhanced efficacy of dust mite sublingual immunotherapy in low-response allergic rhinitis patients after dose increment at 6 months: A prospective study. Int Arch Allergy Immunol. (2020) 181:311–9. doi: 10.1159/000505746. PMID: 32069460

[25] Creticos PS Gunaydin FE Nolte H Damask C Durham SR . Allergen immunotherapy: The evidence supporting the efficacy and safety of subcutaneous immunotherapy and sublingual forms of immunotherapy for allergic rhinitis/conjunctivitis and asthma. J Allergy Clin Immunol Pract. (2024) 12:1415–27. doi: 10.1016/j.jaip.2024.04.034. PMID: 38685477

[26] Zuberbier T Bachert C Bousquet PJ Passalacqua G Walter Canonica G Merk H . GA² LEN/EAACI pocket guide for allergen-specific immunotherapy for allergic rhinitis and asthma. Allergy. (2010) 65:1525–30. doi: 10.1111/j.1398-9995.2010.02474.x. PMID: 21039596

[27] Lin Z Liu Q Li T Chen D Chen D Xu R . The effects of house dust mite sublingual immunotherapy in patients with allergic rhinitis according to duration. Int Forum Allergy Rhinol. (2016) 6:82–7. doi: 10.1002/alr.21657. PMID: 26575696

[28] Novakova SM Novakova PI Yakovliev PH Staevska MT Mateva NG Dimcheva TD . A three-year course of house dust mite sublingual immunotherapy appears effective in controlling the symptoms of allergic rhinitis. Am J Rhinol Allergy. (2018) 32:147–52. doi: 10.1177/1945892418764966. PMID: 29649893

[29] Periasamy N Pujary K Bhandarkar AM Bhandarkar ND Ramaswamy B . Budesonide vs saline nasal irrigation in allergic rhinitis: A randomized placebo-controlled trial. Otolaryngol Head Neck Surg. (2020) 162:979–84. doi: 10.1177/0194599820919363. PMID: 32393099

[30] Gu J Liang ZP Xu W Liu TZ Li ZR Qin G . Quantitative assessment and correlational analysis of subjective and objective indicators in patients with allergic rhinitis. Asia Pac Allergy. (2024) 14:45–55. doi: 10.5415/apallergy.0000000000000141. PMID: 38827256 PMC11142756

[31] Liu B Feng J Hu S . The role of nasal endoscopy in allergic rhinitis and house dust mite sublingual immunotherapy. Int Arch Allergy Immunol. (2021) 182:690–6. doi: 10.1159/000513810. PMID: 34000723

[32] Canonica GW Bousquet J Casale T Lockey RF Baena-Cagnani CE Pawankar R . Sub-lingual immunotherapy: World Allergy Organization position paper 2009. World Allergy Organ J. (2009) 2:233–81. doi: 10.1097/WOX.0b013e3181c6c379. PMID: 23268425 PMC3488881

[33] Wei TT Gao K Tai JH Wei YJ Zhan B . A study of specific immunoglobulin G4 expression in allergic rhinitis and its value in assessing efficacy and in predicting prognosis of sublingual immunotherapy. Kaohsiung J Med Sci. (2025) 41:e12916. doi: 10.1002/kjm2.12916. PMID: 39739782 PMC11724161

[34] Li Y Xiao H Zeng Y Tang Y Zhou L Liu W . Baseline severity and disease duration can predict the response to allergen-specific immunotherapy in allergic rhinitis. Iran J Allergy Asthma Immunol. (2024) 23:52–8. doi: 10.18502/ijaai.v23i1.14953. PMID: 38485909

[35] Shamji MH Kappen JH Akdis M Jensen-Jarolim E Knol EF Kleine-Tebbe J . Biomarkers for monitoring clinical efficacy of allergen immunotherapy for allergic rhinoconjunctivitis and allergic asthma: an EAACI Position Paper. Allergy. (2017) 72:1156–73. doi: 10.1111/all.13138. PMID: 28152201

